# Human Cells Display Reduced Apoptotic Function Relative to Chimpanzee Cells

**DOI:** 10.1371/journal.pone.0046182

**Published:** 2012-09-28

**Authors:** Gaurav Arora, Roman Mezencev, John F. McDonald

**Affiliations:** School of Biology and Parker H. Petit Institute of Bioengineering and Biosciences, Georgia Institute of Technology, Atlanta, Georgia, United States of America; University of Kentucky, United States of America

## Abstract

Previously published gene expression analyses suggested that apoptotic function may be reduced in humans relative to chimpanzees and led to the hypothesis that this difference may contribute to the relatively larger size of the human brain and the increased propensity of humans to develop cancer. In this study, we sought to further test the hypothesis that humans maintain a reduced apoptotic function relative to chimpanzees by conducting a series of apoptotic function assays on human, chimpanzee and macaque primary fibroblastic cells. Human cells consistently displayed significantly reduced apoptotic function relative to the chimpanzee and macaque cells. These results are consistent with earlier findings indicating that apoptotic function is reduced in humans relative to chimpanzees.

## Introduction

Although the human and chimpanzee genomes are highly similar (>98.5% sequence identity) [1], the two species have accumulated significant differences in a number of phenotypic traits since diverging from a common ancestor 6–8 million years ago [2]. Among the most notable of these phenotypic differences is the substantially larger size of the human brain (∼3×) and associated areas of specialized function [3], [4]. A second notable difference between the two species is the significantly higher incidence of cancer in humans relative to chimpanzee (*e.g.*, [5]–[8]) even after adjustment for differences in lifespan [9]. We recently proposed that the evolutionary differences in these traits may, at least in part, be commonly rooted in molecular differences in apoptosis, or programmed cell death [10]. Neurons in the human cerebral cortex are only produced during early development [11]. As neurons in the neocortex are lost over the life span of humans and other primates, they are not replaced. Thus, one potential mechanism by which brain size could have been increased in humans relative to the other primates is by a reduction in the rate of programed cell death. In support of this model, we previously observed that apoptotic pathway genes are differentially expressed between human and chimpanzee brains and other tissues consistent with a generally reduced apoptotic function in humans [10]. Since reduced apoptotic function is well known to be associated with an increased propensity for cancer [5]–[8], we hypothesized that selection for increased cognitive function in humans may have coincidently contributed to an increased propensity for cancer [10].

A key premise of this hypothesis is the validity of the assertion that apoptotic function is indeed reduced in humans relative to chimpanzees. In an initial effort to experimentally test this hypothesis, we conducted a series of experiments designed to detect differences in apoptotic function among human, chimpanzee and macaque (out-group) primary fibroblast cells. The results are uniformly consistent with the hypothesis that apoptotic function is significantly reduced in humans relative to chimpanzees and macaques.

## Results

### Human cells display higher cell viability than chimpanzee and macaque cells after treatment with apoptosis-inducing agents

A number of previously published comparative studies have successfully employed primary fibroblasts to study molecular differences between humans and non-human primates [12], [13]. These previous studies have shown that fibroblasts accurately display molecular properties characteristic of the two species making primary fibroblasts a useful model for inter-specific comparisons. Thus, we decided to use this model to compare relative apoptotic function among human, chimpanzee and macaque cells.

To test for differences in cell viability between human (AG13153), chimpanzee (S006007) and macaque (AG07915) fibroblast cells after induction of apoptosis, cells were treated with the apoptosis-inducing agents staurosporine and mitomycin C (MMC). Following treatment, differences in cell viability between the treated and untreated cells were measured using a cell viability assay.

Staurosporine is a natural product isolated from *Streptomyces staurosporeus*. It induces apoptosis by inhibiting protein kinase C, a known activator of the anti-apoptotic *Bcl-2* gene [14]. MMC is a natural product isolated from *Streptomyces caespitosus* [15] and is a known chemotherapeutic agent used in the treatment of a number of cancers [16]. MMC is a bioreductive DNA alkylating agent that damages DNA via monofunctional and bifunctional adducts (the latter involve cross-linking of guanine bases in the same or adjacent strands of DNA). This cross-linking triggers a powerful apoptotic stimulus, including the activation of p53 [17].

**Figure 1:**
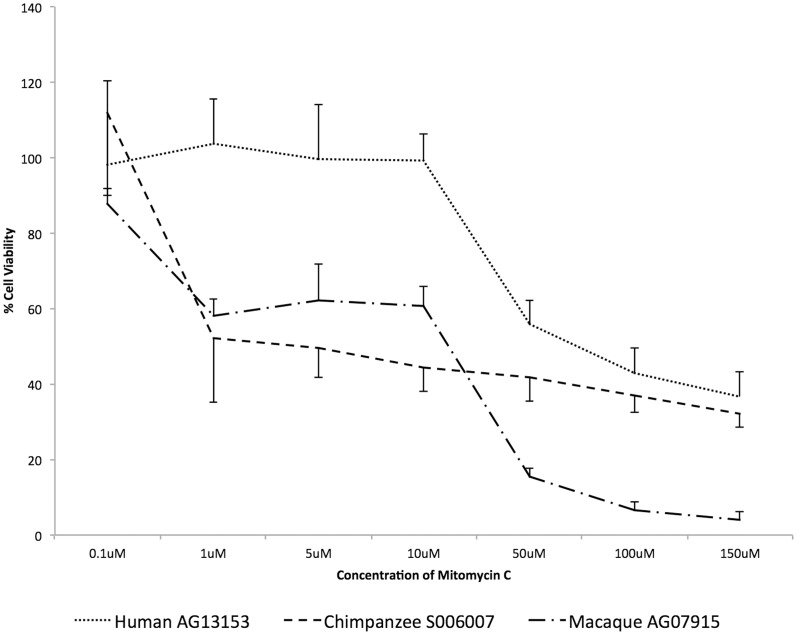
Viability of human, chimpanzee and macaque primary fibroblasts after treatment with MMC. Cell viability (resazurin-based TOX-8 assay) was measured after treatment of cells over a range of concentrations (0.1 µM–150 µM) for 72 hrs. Each data point represents the mean of four independent biological replicates ± SD. Cell viability is expressed as the percentage of the viable cells in the treated group relative to the untreated control group. Differences in cell viability are significant between humans and chimpanzees at 1 µM, 5 µM and 10 µM (p<0.05), and between humans and macaques at all concentrations >0.1 µM of MMC.

Cells derived from each species were treated with MMC, in a concentration dependent manner, and cell viability was measured 72 hours after treatment using the resazurin-based TOX-8 assay. Treating the cells with MMC significantly reduced viability of the chimpanzee (S006007) and macaque (AG07915) cells even at relatively low drug concentrations (1–10 µM), while the human cells (AG13153) displayed reduced viability only at higher concentrations of the drug (50 µM) (Figure 1). The relative cell viability differences between the human and chimpanzee cells were significant (Student's t-test, p<0.05) at 1 µM, 5 µM, and 10 µM of MMC. Likewise, differences between the human and the macaque cells were significant at all concentrations of the drug >0.1 µM of MMC. For all concentrations ≥1.0 µM, cell viability was higher in the human cells relative to chimpanzee and macaque cells consistent with reduced apoptotic function in the humans.

**Figure 2:**
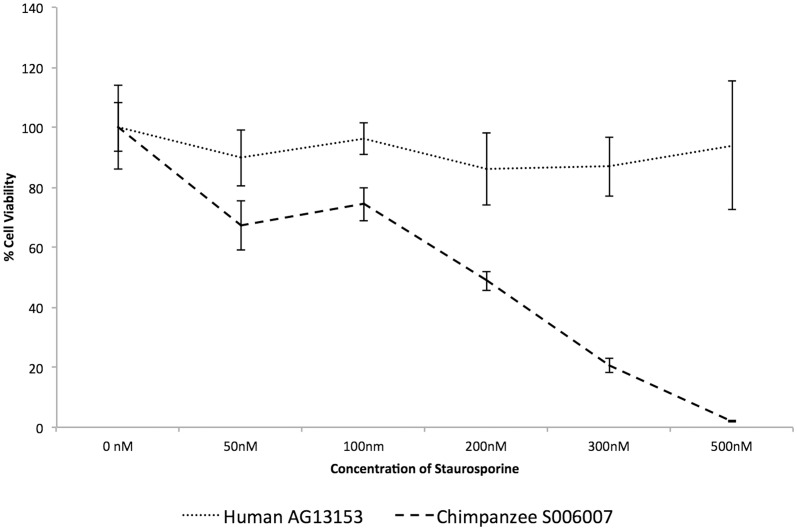
Viability of human and chimpanzee primary fibroblasts after treatment with staurosporine. Cell viability (resazurin-based TOX-8 assay) was measured after treatment of cells over a range of concentrations (50–500 nM) for 48 hrs. Each data point represents the mean ± SD of four independent biological replicates. Cell viability is expressed as the percentage of viable cells in the treated group relative to the untreated controls. The difference in cell viability is significant between humans and chimpanzees at all the concentrations of the drug (Student's t-test, p<0.05).

Similar results were seen when the human (AG13153) and chimpanzee (S006007) cells were treated with staurosporine (Figure 2), with the human cells displaying significantly (Student's t-test, p<0.05) higher viability than the chimpanzee cells at all concentrations of staurosporine.

### Human cells treated with MMC display significantly higher IC_50_ values than chimpanzee or macaque cells

IC_50_ values (the half maximal inhibitory concentrations) reflect the potency of a compound to inhibit growth and viability of cells. The IC_50_ values of MMC were determined and compared among human (AG13153), chimpanzee (S006007) and macaque (AG07915) cells. The results presented in Table 1, show that the MMC IC_50_ values are significantly higher (Student's t-test, p<0.05) for the human cells than either chimpanzee or macaque cells, consistent with the hypothesis that human cells have reduced apoptotic function.

**Table 1:** Relative IC_50_ values after treatment of human, chimpanzee and macaque cells with MMC.

Cell Line	Mean IC50
Human (AG13153)	78.87 µM
Chimpanzee (S006007)	10.72 µM
Macaque (AG07915)	6.04 µM

The differences in IC_50_ values between the human (AG13153) and chimpanzee (S006007) cells and between the human (AG13153) and macaque (AG07915) cells are significant (Student's t-test, p<0.05).

### Human cells display phenotypic features characteristic of reduced apoptotic function relative to chimpanzee and macaque cells after treatment with MMC

**Figure 3:**
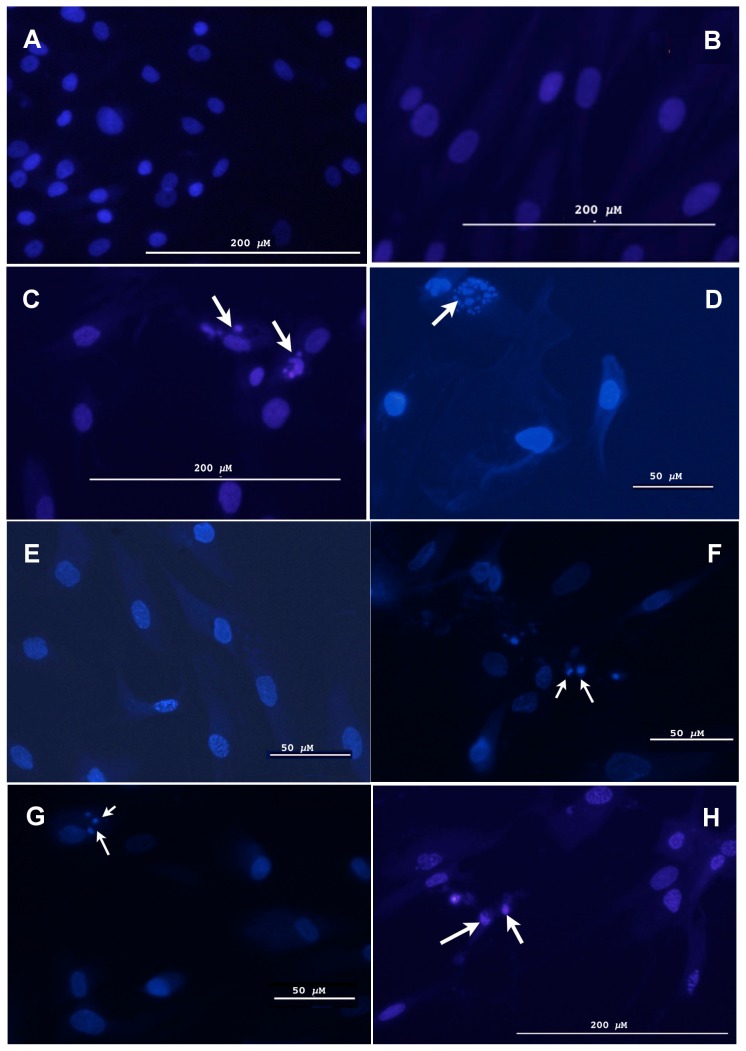
Human cells display morphological features characteristic of reduced apoptotic function relative to chimpanzee and macaque cells after treatment with MMC. Cells treated with MMC for 72 hrs were stained with 10 µg/ml of Hoechst 33342 and visualized under a fluorescence microscope. More than 100 cells were examined in 4 separate views (>25 cells per view). The pictures displayed are representative of the overall results. (A) Chimpanzee cells (S006007) untreated (note: untreated human cells display the same phenotype as untreated chimpanzee cells); B) Human cells (AG13153) treated with 10 µM of MMC; C) Chimpanzee cells (S006007) treated with 10 µM of MMC; D) Macaque cells (AG07915) treated with 10 µM of MMC; E) Human cells (AG07307) treated with 15 µM of MMC; F) Chimpanzee cells (S005795) treated with 15 µM of MMC; G) Macaque cells (AG07128) treated with 15 µM of MMC; and H) Human cells (AG13153) treated with 100 µM of MMC. Arrows indicate nuclei displaying an apoptotic phenotype (*i.e.*, chromatin fragmentation/condensation, C, D, F, G and H). Control cells (A) display a normal cellular phenotype.

Cell viability may be affected by apoptosis, necrosis, autophagy, pyroptosis or mitotic catastrophe [18]. To confirm that the observed differences in viability were due to apoptosis, the morphological features of the cell nuclei were examined. Cells were treated over a range of concentrations of MMC (10 µM, 15 µM and 100 µM) for 72 hrs, washed with PBS, fixed and then stained with 10 µg/ml of Hoechst 33342 for 15 minutes. The cells were then visualized under a fluorescent microscope for features characteristic of apoptosis (Figure 3A–H).

The nuclei of the untreated control cells had an oval shape with homogeneous intensity (Figure 3A). Cells treated with MMC in which apoptosis has been induced typically display condensed and fragmented shapes of nuclei with irregular staining patterns [19]. The phenotypic characteristics of both cultures of human cells treated with 10 µM (Figure 3B) and 15 µM (Figure 3E) of MMC, respectively, were similar to those of the untreated control cells (Figure 3A). Only at the higher concentration of MMC (100 µM), did the human cells show phenotypic characteristics of apoptosis (Figure 3H).

In contrast to human cells, the chimpanzee cells treated with low concentrations (10 µM -Figure 3C) and 15 µM -Figure 3F) of MMC displayed chromatin condensation and fragmentation consistent with apoptosis. Similar results were observed for the macaque cells treated with 10 µM (Figure 3D) and 15 µM (Figure 3G) of MMC. Our analysis of gene expression data [12] indicates that the response of human and chimpanzee fibroblasts to MMC cannot simply be attributed to changes in expression of genes previously associated with MMC activation, detoxification, transport and repair of MMC-induced DNA damage on mRNA level (see File S1). Collectively, our results are consistent with the hypothesis that apoptotic function is reduced in human cells relative to those of chimpanzee and macaque.

### Human cells display lower caspase-3/7 activity than chimpanzee cells after treatment with staurosporine

**Figure 4:**
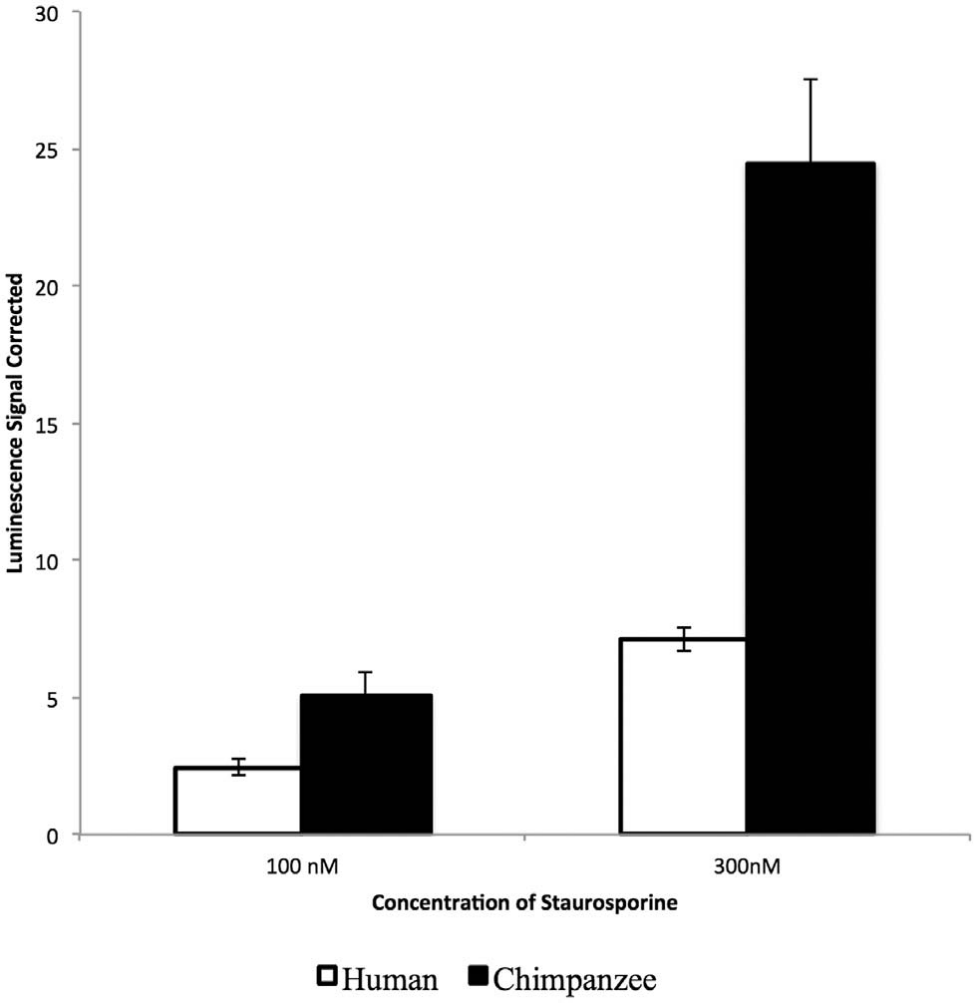
Human cells display lower caspase-3/7 activity than chimpanzee cells after treatment with staurosporine. Histograms display the mean of three independent biological replicates ± SD for each species. Chimpanzee cells (S006007) displayed significantly higher caspase-3/7 activity than human cells (AG13153) at 100 nM of staurosporine (Student's t-test, p = 0.023) and at 300 nM of staurosporine (Student's t-test, p = 0.011).

The executioner caspase-3 and caspase-7 proteases are activated during apoptosis and are considered a biomarker of the process [20]. The activity of these caspases was compared between human (AG13153) and chimpanzee (S006007) cells after treatment with increasing concentrations (100 nM and 300 nM) of the apoptosis-inducing drug staurosporine using the Caspase-3/7 Glo Assay [21]. For both concentrations of the drug, the chimpanzee cells displayed significantly (Student's t-test, p<0.03) higher caspase-3/7 activity than the human cells (Figure 4), consistent with the hypothesis that apoptotic function is reduced in human cells.

### Human cells display reduced dissipation of mitochondrial transmembrane potential relative to chimpanzee cells after treatment with MMC

The apoptotic pathway consists of the extrinsic and the intrinsic pathways [20], both of which converge on activating the executioner caspases, caspase-3 and caspase-7 [22]. The intrinsic pathway proceeds through the release of pro-apoptotic factors (cytochrome C, Diablo and apoptosis-inducing factor) present in the mitochondrial intermembrane space, which then leads to the activation of apoptosis. This release of pro-apoptotic factors occurs as a result of the permeabilization of the inner and outer mitochondrial membranes [23].

Under normal physiological conditions, a transmembrane electrical potential gradient (ΔΨ_m_) is maintained across the inner mitochondrial membrane, and this gradient is indicative of the normal functioning of the mitochondria [24]. On the induction of apoptosis, the mitochondrial transmembrane potential collapses [25]. Reduction of ΔΨ_m_ is indicative of apoptosis and can be determined via decreased fluorescence using lipophilic, cationic fluorescent dyes, such as tetramethylrhodamine, ethyl ester (TMRE) [26].

TMRE was used to test whether the proportion of apoptotic cells differed between MMC-treated human and chimpanzee fibroblasts. Human (AG07307) and chimpanzee (S005795) cells were treated with 30 µM and 100 µM of MMC. After 72 hours, the cells were stained with 100 nM of TMRE dye (MitoPT TMRE Assay Kit, Immunochemistry, Bloomington, MN), and the distribution of TMRE fluorescence intensity of MMC-treated and untreated control cells was analyzed by flow cytometry.

**Figure 5:**
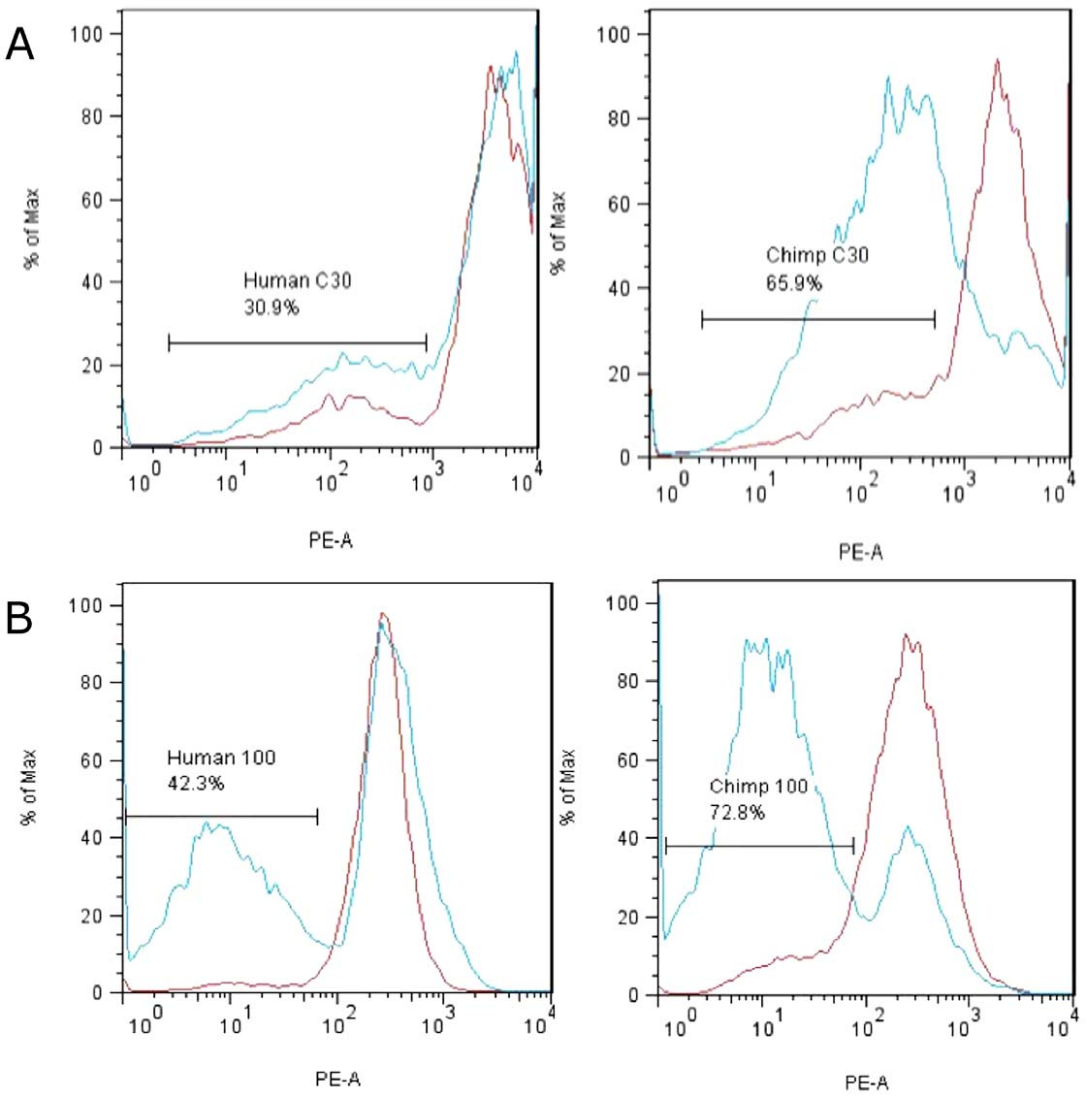
Mitochondrial transmembrane potential (ΔΨ_m_) of human and chimpanzee cells. Human (AG07307), and chimpanzee (S005795) cells were treated with 30 µM (A) or 100 µM (B) of MMC and mitochondrial membrane potential determined by monitoring fluorescence of TMRE (tetramethyl rhodamine, ethyl ester). A reduction in fluorescence is indicative of dissipation of mitochondrial transmembrane potential and the onset of apoptosis. The results indicate that MMC-treated chimpanzee cells display higher proportion of apoptotic cells than human cells (Red lines = untreated cells; Blue lines = cells treated with the indicated concentrations of MMC; x-axis = PE-A channel, y-axis = normalized cell number).

Following treatment with 30 µM of MMC, 65.9% of chimpanzee cells displayed dissipated mitochondrial membrane potential (ΔΨ_m_) relative to only 30.9% of human cells (p<0.05, Figure 5A). Similarly, after treatment with 100 µM of MMC, 72.8% of chimpanzee cells displayed dissipated mitochondrial membrane potential relative to 42.3% displayed by human cells (p<0.05, Figure 5B). The results indicate that apoptotic function is significantly reduced in human relative to chimpanzee cells.

## Discussion

The fact that the apoptotic process plays a key role in both brain development and cancer progression, led us to previously hypothesize that the differences in brain size and propensity for cancer that exists between humans and chimpanzees may both be associated, at least in part, with differences in apoptotic function [10]. In agreement with this hypothesis, we previously observed that differences in the expression of apoptotic pathway genes between human and chimpanzee brains and other tissues were consistent with reduced apoptotic function in humans [10]. In this study, we sought to further test the hypothesis that apoptotic function is reduced in humans relative to chimpanzees by monitoring the relative response of human and chimpanzee primary fibroblast cells to apoptotic-inducing agents.

A variety of assays were conducted using 6 independently established primate primary cell cultures (2 human, 2 chimpanzee and 2 macaque) and the results were uniformly consistent with the hypothesis that the apoptotic function in humans is significantly reduced relative to chimpanzees and macaques. At low concentrations of the apoptosis-inducing agent MMC (1–10 µM and 15 µM), chimpanzee cells displayed a significantly lower number of viable cells compared to the human samples. The nuclear morphology of the chimpanzee cells at these concentrations was typical of apoptosis showing nuclear condensation and fragmentation. In contrast, the nuclear morphology of the human cells at these low concentrations was similar to untreated cells (Figure 3A), indicating that very little or no apoptosis was occurring in the human samples. Comparisons of the IC_50_ values of MMC treated human and chimpanzee cells confirmed that the chimpanzee cells were more sensitive to MMC-induced apoptosis. Although further testing will be required before definitive conclusions can be drawn, our results are consistent with the hypothesis that humans maintain a reduced apoptotic function relative to chimpanzees.

To determine whether this putative difference in apoptotic function between humans and chimpanzees is most likely to have occurred in the human or chimpanzee lineage, we used macaque as an out-group in our assays. The results consistently indicated that macaque cells behaved similarly to chimpanzee cells in displaying higher sensitivity to apoptosis-inducing agents relative to human cells. These findings suggest that the reduced apoptotic function associated with human cells is an evolutionarily derived condition occurring within the human lineage subsequent to the divergence of humans and chimps from a common ancestor ∼6 MYA. Consistent with this view are previous findings indicating that apoptotic genes display accelerated rates of evolutionary change within the human lineage relative to the other primates [27].

## Materials and Methods

### Fibroblast Cells

Primary human, chimpanzee and macaque skin fibroblast cells were obtained from Coriell cell repositories (Camden, NJ, USA). Human cells: AG13153 – established from a 30-year-old male; AG07307 – established from a 40-year-old female; Chimpanzee cells: S006007 – established from a 22-year-old male; S005795 – established from a 26-year-old female; Macaque cells: AG07915 - established from a 12-year-old male; AG07128 – established from an 11-year-old female.

### Cell Culture

Human primary fibroblasts were cultured in Minimum Essential Medium with Hank's BSS supplemented with 2 mM L-glutamine (Mediatech, VA, USA), antibiotic/antimyotic (100 IU/ml Penicillin, 100 µg/ml Streptomycin, 0.25 µg/ml Amphotericin B, Mediatech, VA, USA), 26 mM Hepes (Sigma, MO, USA) and 10% FBS (Gibco, NY, USA) at 37°C in humidified air with 5% CO_2_. Chimpanzee primary fibroblasts were cultured in Minimum Essential Medium alpha-modification with nucleosides with 2 mM L-glutamine (Mediatech, VA, USA), antibiotic/antimyotic (100 IU/ml Penicillin, 100 µg/ml Streptomycin, 0.25 µg/ml Amphotericin B, Mediatech, VA, USA) and 10% FBS (Gibco, NY, USA) at 37°C in humidified air with 5% CO_2_ . Macaque primary fibroblasts were cultured in Minimum Essential Medium alpha-modification with nucleosides with 2 mM L-glutamine (Mediatech, VA, USA), antibiotic/antimyotic (100 IU/ml Penicillin, 100 µg/ml Streptomycin, 0.25 µg/ml Amphotericin B, Mediatech, VA, USA) and 15% FBS (Gibco, NY, USA) at 37°C in humidified air with 5% CO_2_. The growth rates for each of the cells were determined in 96 well plates using the TOX-8 (Sigma-Aldrich, St. Louis, MO, USA) cell cytotoxicity assay. Passage numbers at which tests were done were as follows: human (AG13153) P14–P16; chimpanzee (S006007) P14–P16; macaque (AG07915) P16–P18; human (AG07307) P17–P19; chimpanzee (S005795) P18–P20; and macaque (AG07128) P18–P20.

### Cell Viability Assay

Mitomycin C was obtained from Sigma Aldrich, MO, USA, and staurosporine from Fisher Scientific, PA, USA. The cell viability experiments were conducted in 96 well plates using the resazurin-based In Vitro Toxicology Assay Kit TOX-8 (Sigma-Aldrich, St. Louis, MO, USA) as previously described [28]. For each cell type, the number of cells seeded per well (as determined by pre-test optimization) was 80,000 cells/ml for the human cells, 120,000 cells/ml for the chimpanzee cells and 60,000 cells/ml for the macaque cells. The cells were treated over a range of concentrations of staurosporine and MMC for 48 and 72 hours respectively in RPMI growth medium supplemented with 5% FBS and penicillin (100 IU/mL), streptomycin (100 µg/mL) and amphotericin B (0.25 µg/mL).

### Hoechst Staining

The cells in the 96 well plates treated with MMC for 72 hours and untreated control cells were washed twice with 100 µl of PBS, fixed with 10% buffered formal-saline for 30 minutes and stained with 10 µg/ml of Hoechst 33342 (Sigma-Aldrich) in H_2_O for 15 minutes. The cells were then visualized using a fluorescence microscope (Olympus IX51, Olympus, NJ, USA) and photographed using an Olympus DP72 digital camera.

### Caspase-3/7 Activity Assay

The human (AG13153) and chimpanzee (S006007) cells were allowed to grow for 24 hours in white walled cell culture-treated 96 well plates, and with staurosporine for 48 hours to induce apoptosis. Following treatment with the drug, 100 µl of the Caspase-3/7 Glo reagent (Promega Corporation, WI, USA) was added, followed by incubation at room temperature for 30 minutes to generate a luminescence signal. The caspase-3/7 activities were determined by measuring luminescence signal using a microplate reader (Spectramax Gemini XS, Molecular Devices, CA, USA).

### Measurement of Mitochondrial Transmembrane Potential

Changes in ΔΨ_m_ upon treatment with MMC were detected by flow cytometry experiment using tetramethylrhodamine ethyl ester (TMRE). Human (AG07307) and chimpanzee (S005795) cells were grown in 100 mm Petri dishes for 24 hours and subsequently treated with different concentrations of MMC for 72 hours. Thereafter, adherent cells were harvested by trypsinization, combined with free-floating cells in growth medium and stained in dark with 100 nM of TMRE for 30 minutes at 37°C. TMRE fluorescence was measured using the PE channel of the BD LSR II flow cytometer (BD Biosciences, NJ, USA). Analysis of the data was carried out using FlowJo 7.6 software (Tree Star, Inc., Ashland, OR, USA).

### Statistical Analysis

When not specified otherwise, significance of differences between means was tested by two-tailed Student's t-test. Differences in proportions of cells with dissipated ΔΨ_m_ between MMC-treated cells were tested using two-proportion z-test. IC_50_ values were determined by non-linear regression of log-transformed data using a normalized response-variable slope model (GraphPad Prism 5.01; GraphPad Software, Inc.).

## Supporting Information

File S1 Differential expression between human and bonobo fibroblasts of genes previously shown to be associated with resistance to MMC.
(DOCX)
