# Supplementary material for: Human Cells Display Reduced Apoptotic Function Relative to Chimpanzee Cells
Source: PLoS One. 2012 Sep 28;7(9):e46182. doi: 10.1371/journal.pone.0046182 (PMC3460825; doi:10.1371/journal.pone.0046182)
Supplement: File S1 — Differential expression between human and bonobo fibroblasts of genes previously shown to be associated with resistance to MMC. (DOCX) [file pone.0046182.s001.docx]

**Human Cells Display Reduced Apoptotic Function Relative to Chimpanzee Cells**

Gaurav Arora^[[1]](#footnote-1)^, Roman Mezencev^1^ and John F. McDonald^1^*

^1^School of Biology, Georgia Institute of Technology, Atlanta, GA 30332

*Corresponding author

Email: john.mcdonald@biology.gatech.edu

**Supplemental Information File S1**

The resistance of various human cells to mitomycin C (MMC) has been previously associated with decreased expression of genes involved in the conversion of MMC into an active drug (*NQO1, POR*) and/or increased expression of genes involved in glutathione metabolism (*GSTP1, GSR, GPX1, GCLC, GCLM*), nucleotide excision and homologous recombination repair (*ERCC1, XDH*) or multidrug resistance (*ABCB1*) (See below).

To investigate the possibility, that other than apoptosis-related mechanisms were responsible for the observed higher cell viability and lower proportion of apoptotic cells in MMC-treated human cells relative to chimpanzee cells, we compared expression of these genes using repository U95Av2 microarray data for 18 human and 10 bonobo primary fibroblast cultures [1]. At the level of multiplicity adjusted p-value < 0.05, none of the genes known to confer resistance to mitomycin C, except for *GSR*, demonstrated higher expression levels in human compared to bonobo cells (below).

**Differential expression between human and bonobo fibroblasts of genes previously shown to be associated with resistance to MMC**. Microarray data were obtained from Karaman et al. [1]. Probe ID corresponds to U95Av2 3’-expression arrays (Affymetrix). FC (fold change of expression value between humans and bonobo; p-values were multiplicity adjusted using the Benjamini-Hochberg method [2].

| **Gene symbol** | **Probe ID** | **FC (h/b)** | **Adjusted p-value** | **Ref** |
| --- | --- | --- | --- | --- |
| ERCC1 | 1878_g_at | -1.77 | 0.0028 | [3] |
| GSTP1 | 33396_at | -1.36 | 0.0153 | [4] |
| GSR | 35130_at | 1.24 | 0.036 | [4] |
| POR | 858_at | 1.59 | 0.0702 | [5,6] |
| GPX1 | 37033_s_at | -1.29 | 0.3195 | [4] |
| GCLM | 33163_r_at | -1.28 | 0.3754 | [7] |
| XDH | 33463_at | -1.15 | 0.4693 | [6] |
| GCLC | 31850_at | -1.13 | 0.6348 | [7] |
| ABCB1 | 1682_s_at | -1.01 | 0.6 | [8] |
| NQO1 | 38066_at | -1.02 | 0.9527 | [9,10] |

Similarly, genes activating MMC were not differentially expressed between human and bonobo fibroblastic cells. The *GSR* (glutathione reductase) gene was overexpressed in human cells; however, the low fold change value (1.24) and the fact that 6 out of 16 probes in probeset “35130_at” do not perfectly match with the target chimpanzee sequence (below) suggests that the role of GSR in lower susceptibility of human fibroblasts to MMC is negligible.

**Probeset ID: 35130_at ( human glutathione reductase, GSR) alignment with chimpanzee sequences from the *Pan troglodytes* genome.** Probe information (top line each box) retrieved from NetAffx (http://www.affymetrix.com/analysis/index.affx) and chimp sequence (bottom line each box). Green color: perfect match; red color: imperfect match.

| Probe sequence 5’3’  Probe-target alignment |
| --- |
| tcccgaataccaaggacctgagttt  \|\|\|\|\|\|\|\|\|\|\|\|\|\|\|\|\|\|\|\|\|\|\|\|\|  tcccgaataccaaggacctgagttt |
| caaactggggattcaaaccgatgac  \| \|\|\|\|\|\|\|\|\|\|\|\|\|\|\|\|\|\|\|  aatttaggggattcaaaccgatgac |
| aagggtcatatcatcgtagacgaat  \|\|\|\|\|\|\|\|\|\|\|\|\|\| \|\|\|\|\|\|\|\|\|\|  aagggtcatatcattgtagacgaat |
| tcatcgtagacgaattccagaatac  \|\|\|\| \|\|\|\|\|\|\|\|\|\|\|\|\|\|\|\|\|\|\|\|  tcattgtagacgaattccagaatac |
| gattataacaacatcccaactgtgg  \|\|\|\|\|\|\|\|\|\|\|\|\|\|\|\|\|\|\|\|\|\|\|\|\|  gattataacaacatcccaactgtgg |
| acatcccaactgtggtcttcagcca \|\|\|\|\|\|\|\|\|\|\|\|\|\|\|\|\|\|\|\|\|\|\|\|\|  acatcccaactgtggtcttcagcca |
| atgtgaagacctattcaacgagctt  \|\|\|\|\|\|\|\|\|\|\|\|\|\|\|\|\|\|\|\|\|\|\|\|\|  atgtgaagacctattcaacgagctt |
| atgtatcacgcagttaccaaaagga  \|\|\|\|\|\|\|\|\|\|\|\|\|\|\|\|\|\|\|\|\|\|\|\|\| atgtatcacgcagttaccaaaagga |
| aatgctgcagggttttgctgttgca  \|\|\|\|\|\|\|\|\|\|\|\|\|\|\|\|\|\| \|\|\|\|\|\|  aatgctgcagggttttgccgttgca |
| tgctgcagggttttgctgttgcagt  \|\|\|\|\|\|\|\|\|\|\|\|\|\|\|\| \|\|\|\|\|\|\|\|  tgctgcagggttttgccgttgcagt |
| ctgcagggttttgctgttgcagtga  \|\|\|\|\|\|\|\|\|\|\|\|\|\| \|\|\|\|\|\|\|\|\|\|  ctgcagggttttgccgttgcagtga |
| caccctacctcttcagaagagctgg  \|\|\|\|\|\|\|\|\|\|\|\|\|\|\|\|\|\|\|\|\|\|\|\|\| caccctacctcttcagaagagctgg |
| cctacctcttcagaagagctggtca \|\|\|\|\|\|\|\|\|\|\|\|\|\|\|\|\|\|\|\|\|\|\|\|\|  cctacctcttcagaagagctggtca |
| gcgggcagtgggacccatagatctt \|\|\|\|\|\|\|\|\|\|\|\|\|\|\|\|\|\|\|\|\|\|\|\|\|  gcgggcagtgggacccatagatctt |
| cagtgggacccatagatcttctgaa  \|\|\|\|\|\|\|\|\|\|\|\|\|\|\|\|\|\|\|\|\|\|\|\|\| cagtgggacccatagatcttctgaa |
| gacccatagatcttctgaaatgaaa  \|\|\|\|\|\|\|\|\|\|\|\|\|\|\|\|\|\|\|\|\|\|\|\|\| gacccatagatcttctgaaatgaaa |

**References:**

1. Karaman MW, Houck ML, Chemnick LG, Nagpal S, Chawannakul D, et al (2003) Comparative analysis of gene-expression patterns in human and African great ape cultured fibroblasts. Genome Res 13(7): 1619-1630.

2. Benjamini Y, Yekutieli D (2001) The control of the false discovery rate in multiple testing under dependency. Annals of Stat 29 (4): 1165–1188.

3. Cummings M, Higginbottom K, McGurk CJ, Wong OG, Köberle B, et al (2006) XPA versus ERCC1 as chemosensitising agents to cisplatin and mitomycin C in prostate cancer cells: role of ERCC1 in homologous recombination repair. Biochem Pharmacol 72(2): 166-75.

4. Perry RR, Kang Y, Greaves B (1993) Biochemical characterization of a mitomycin C

resistant colon cancer cell line variant. Biochem Pharmacol 46(11): 1999-2005. PubMed PMID: 7903534.

5. Hoban PR, Walton MI, Robson CN, Godden J, Stratford IJ, et al (1990) Decreased NADPH:cytochrome P-450 reductase activity and impaired drug activation in a mammalian cell line resistant to mitomycin C under aerobic but not hypoxic conditions. Cancer Res 50(15): 4692-7. PubMed PMID:2114946.

6. Pan SS, Andrews PA, Glover CJ, Bachur NR (1984) Reductive activation of mitomycin C and mitomycin C metabolites catalyzed by NADPH-cytochrome P-450 reductase and xanthine oxidase. J Biol Chem 259(2): 959-66. PubMed PMID: 6319393.

7. Walsh AC, Feulner JA, Reilly A (2001) Evidence for functionally significant

polymorphism of human glutamate cysteine ligase catalytic subunit: association

with glutathione levels and drug resistance in the National Cancer Institute

tumor cell line panel. Toxicol Sci 61(2): 218-23. PubMed PMID: 11353130.

8. Hayes MC, Birch BR, Cooper AJ, Primrose JN (2001) Cellular resistance to mitomycin C is associated with overexpression of MDR-1 in a urothelial cancer cell line (MGH-U1). BJU Int 87(3): 245-50. PubMed PMID: 11167651.

9. Xu BH, Gupta V, Singh SV (1994) Characterization of a human bladder cancer cell line selected for resistance to mitomycin C. Int J Cancer 58(5): 686-92.

PubMed PMID: 8077054.

10. Lambert PA, Kang Y, Greaves B, Perry RR (1998) The importance of DT-diaphorase in mitomycin C resistance in human colon cancer cell lines. J Surg Res 80(2): 177-81. PubMed PMID: 9878310.

1. [↑](#footnote-ref-1)
